# Phenolic Composition, Antioxidant Activity and Caffeine as Chemical Markers for Differentiating Panamanian *Coffea arabica* and *Coffea canephora*

**DOI:** 10.3390/molecules31091534

**Published:** 2026-05-05

**Authors:** Mariel Monrroy, Onix Arauz, José Renán García

**Affiliations:** 1 Research Center in Biochemistry and Applied Chemistry, Faculty of Natural and Exact Sciences, Autonomous University of Chiriqui, David 04001, Panama; 2 Department of Chemistry, Faculty of Natural and Exact Sciences, Autonomous University of Chiriqui, David 04001, Panama; 3 National Research System (SNI), National Secretariat of Science, Technology and Innovation (SENACYT), Panama City 0816-02852, Panama

**Keywords:** *Coffea arabica*, *Coffea canephora*, phenolic compounds, antioxidant activity, caffeine, response surface methodology, chemometrics

## Abstract

Chemical differentiation of coffee species is essential for quality control, authenticity verification, and the prevention of mislabeling in the coffee industry. This is particularly relevant in Panama, a recognized producer of specialty coffees such as Geisha, where studies on chemical characterization remain limited. This study investigated the phenolic composition, antioxidant activity, and caffeine content of roasted coffee as potential chemical markers for differentiating *Coffea arabica* and *Coffea canephora*. Ultrasound-assisted hydroethanolic extraction was optimized using the response surface methodology, with temperature, ethanol concentration, and extraction time as the experimental variables. The optimized extraction parameters were established at 75 °C, 44% ethanol, and 25 min, resulting in high recovery of the total phenolic content, total flavonoid content, and antioxidant activity (ABTS and DPPH assays). Under these conditions, *C. canephora* samples obtained higher levels of phenolic compounds, antioxidant activity, and caffeine content than *C. arabica*. Pearson’s correlation analysis showed significant associations among phenolic compounds, caffeine content, and antioxidant activity. PCA explained 90.5% of the total variance and clearly discriminated between the two species, mainly based on differences in caffeine content and antioxidant activity. HCA confirmed this classification and revealed subgroups within *C. arabica*, particularly among the Geisha varieties. PLS-DA achieved complete separation between species, with zero classification error under cross-validation. These results indicated that combined chemical and multivariate approaches can be used to differentiate and assess the authenticity of coffee, particularly in underexplored production regions such as Panama.

## 1. Introduction

Coffee is one of the most widely consumed beverages worldwide, and its production and commercialization play a major role in the global economy [1,2]. According to the International Coffee Organization, global coffee production reached approximately 168.2 million 60 kg bags in the 2021/2022 season. The coffee market is dominated by two species—namely, *Coffea arabica* (56%) and *Coffea canephora* (also known as Robusta, 44%)—with Brazil, Vietnam, and Colombia as the leading producers [3]. Although Panama contributes a relatively small share to global coffee production, it has gained international recognition for the exceptional quality of its specialty coffee, particularly high-end *C. arabica* varieties.

Among coffee species, *C. arabica* has a higher commercial value owing to its superior sensory attributes, particularly its aroma and flavor. This high market value has made *C. arabica* particularly vulnerable to adulteration, including the addition of coffee husks, cereals, and legumes, or blending with the lower-quality *C. canephora* [4]. At least 29 different adulterants have been reported in roasted and ground coffee products [4]. Roasted and ground coffee is susceptible to fraud because adulteration is difficult to detect visually, which can result in economic deception and reductions in the nutritional quality and bioactive compound content, as well as potential health risks associated with allergenic components [5,6].

Coffee is rich in bioactive compounds, including phenolics, flavonoids, and caffeine, which are largely responsible for its antioxidant capacity and health-related properties. The concentrations of these compounds are strongly influenced by the coffee species, variety, and processing conditions. In general, *C. canephora* contains higher levels of caffeine and phenolic compounds than *C. arabica* [7,8], making these constituents valuable chemical markers for species differentiation and authenticity assessment [9,10].

Several analytical techniques have been employed for coffee characterization and authenticity evaluation, including nuclear magnetic resonance (NMR), chromatography-based methods (GC and HPLC), UV–Vis spectroscopy, thermogravimetric analysis (TGA), and elemental analysis [11,12,13,14,15,16]. Among these approaches, chromatographic techniques such as HPLC are particularly relevant for the accurate quantification of caffeine. In contrast, UV–Vis spectrophotometric methods are widely used to determine the total phenolic and flavonoid contents and antioxidant capacity in coffee matrices.

The Panamanian coffee sector contributes approximately USD 212.2 million to the national economy, with an annual production of approximately 200,000 metric tons [17]. Despite the economic and cultural importance of coffee production in Panama, systematic studies integrating chemical characterization with multivariate analysis for species differentiation remain limited. In particular, region-specific datasets combining phenolic composition, antioxidant activity, and caffeine content are scarce, highlighting the need for comprehensive analytical approaches tailored to Panamanian coffee. Furthermore, although regulatory frameworks strictly prohibit the commercialization of adulterated roasted and ground coffee [18], the lack of standardized analytical protocols tailored to local coffee varieties hinders quality control and authenticity verification.

Therefore, this study aimed to investigate the chemical differences between roasted Panamanian *C. arabica* and *C. canephora* coffee samples by evaluating their phenolic composition, antioxidant activity, and caffeine content. We hypothesized that the integrated evaluation of phenolic composition, antioxidant activity, and caffeine content, combined with multivariate statistical analysis, will enable reliable discrimination between coffee species despite variability associated with processing conditions. Ultrasound-assisted extraction conditions were optimized using the response surface methodology, and the resulting chemical data were analyzed using multivariate statistical analyses. This work contributes to the development of an integrated analytical framework for Panamanian coffee and supports the identification of compositional indicators for authenticity assessment and quality control.

## 2. Results

### 2.1. Optimization of Phenolic Compounds and Antioxidant Activity by Response Surface Methodology

The experimental design matrix and the corresponding responses for total phenolic content (TPC), total flavonoid content (TFC), and antioxidant activity, as measured via 2,2-diphenyl-1-picrylhydrazyl (DPPH) and 2,2′-azinobis-(3-ethylbenzothiazoline-6-sulfonic acid) (ABTS) assays under different extraction conditions, are shown in Table 1. Quadratic polynomial models were fitted to describe the effects of temperature, ethanol concentration, and extraction time on the responses. The fitted response surface equations (Equations (1)–(4)) were evaluated using analysis of variance (ANOVA).

(1) $$TPC\left(mg\;GA\;g^{-1}\right):=48.7\pm 3.3+5.9\pm 1.9X_{1}-1.9\pm 1.9X_{2}+4.0\pm 1.9X_{3}-4.0\pm 2.6{X_{2}}^{2}-3.3\pm 2.6{X_{3}}^{2}$$

(2) $$TFC\left(mg\;QE\;g^{-1}\right):=23.3\pm 0.8+2.8\pm 0.5X_{1}-1.2\pm 0.5X_{2}+1.4\pm 0.5X_{3}-1.0\pm 0.7{X_{2}}^{2}-1.0\pm 0.7{X_{3}}^{2}-0.7\pm 0.6X_{1}X_{3}+0.8\pm 0.6X_{2}X_{3}$$

(3) $$ABTS\left(µmoles\;TE\;g^{-1}\right):=241.1\pm 8.3+28.5\pm 4.7X_{1}-7.7\pm 4.7X_{2}+20.0\pm 4.7X_{3}-14.9\pm 6.2{X_{1}}^{2}-14.8\pm 6.2{X_{2}}^{2}-11.0\pm 6.2{X_{3}}^{2}$$

(4) $$DPPH\left(µmoles\;TE\;g^{-1}\right):=199.5\pm 7.8+21.1\pm 5.2X_{1}-13.9\pm 5.2X_{2}+15\pm 5.2X_{3}-21.1\pm 6.8{X_{2}}^{2}-8.6\pm 6.8{X_{3}}^{2}$$

In Equations (1)–(4), X_1_ is the temperature, X_2_ is the ethanol content (%), and X_3_ is the time (min). GA refers to gallic acid equivalents, QE to quercetin equivalents, and TE to Trolox equivalents. The coefficients are expressed as estimates ±95% confidence intervals.

The linear terms yielded positive coefficients for temperature and extraction time, indicating that the responses increased with increasing values of these variables up to a maximum level, as indicated by the negative quadratic coefficients. In contrast, ethanol concentration exhibited negative linear coefficients in most models, and lower responses were observed at higher ethanol concentrations. Among the evaluated variables, temperature showed the largest positive linear coefficient in all models (*p* < 0.05), indicating that it exerted the strongest influence on extraction efficiency.

The fitted model parameters are summarized in Table 2. The coefficients of determination (R^2^ = 0.95–0.99) and lack-of-fit (*p* = 0.358–0.787) confirmed the adequacy of the developed models. In addition, the response surface methodology (RSM) models were statistically significant (*p* < 0.001), demonstrating the reliability of the fitted equations. The relatively low relative standard deviation (RSD) values (0.82–8%) further indicate the precision of the experimental data and the suitability of the RSM models for describing the extraction process.

**Figure 1:**
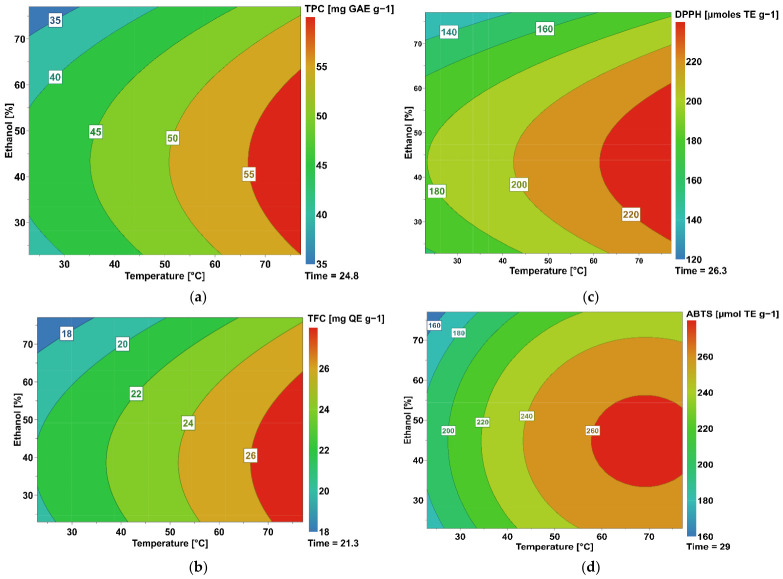
Contour plots showing the combined effects of temperature and ethanol concentration on (**a**) TPC, (**b**) TFC, (**c**) DPPH, and (**d**) ABTS during ultrasound-assisted hydroethanolic extraction. Each graph shows the optimal extraction time predicted by the model for the corresponding variables.

Contour plots illustrating the interactions between the extraction variables and their effects on the response variables are shown in Figure 1.

### 2.2. Determination of Optimal Extraction Conditions for Phenolic Compounds and Antioxidant Activity

The optimal extraction conditions for each response variable were determined individually via numerical optimization using response surface models. The predicted optimal conditions were 76.3 °C, 43.3% ethanol, and 24.8 min for TPC; 76.6 °C, 42.2% ethanol, and 21.3 min for TFC; 75.7 °C, 43.4% ethanol, and 26.3 min for DPPH; and 68.9 °C, 44.8% ethanol, and 29.1 min for ABTS. Under these conditions, the predicted maximum values were 57.8 mg GAE g^−1^ DW (TPC), 27.4 mg QE g^−1^ DW (TFC), 235.2 µmol TE g^−1^ DW (DPPH), and 264.9 µmol TE g^−1^ DW (ABTS), whereas the experimentally obtained values were 56 ± 1, 31.2 ± 0.8, 225 ± 3, and 240 ± 1, respectively. These results demonstrated good agreement between the predicted and experimental values.

Although the numerical optima differed slightly among responses, all were located within a relatively narrow region characterized by minimal variation in the predicted responses. This indicates that the response surfaces were stable around the optimum region, and small changes in temperature, ethanol concentration, or extraction time did not substantially affect extraction performance. Therefore, a single set of extraction conditions (75 °C, 44% ethanol, and 25 min) was selected within this region as a practical compromise, enabling the simultaneous extraction and determination of all responses using a single procedure. Under these selected conditions, the predicted values were 57.7 mg GAE g^−1^ DW (TPC), 27.0 mg QE g^−1^ DW (TFC), 234.2 µmol TE g^−1^ DW (DPPH), and 261.7 µmol TE g^−1^ DW (ABTS), whereas the experimentally obtained values were 57.4 ± 0.8, 30.8 ± 0.5, 227 ± 2, and 241 ± 1, respectively. The relative deviations between predicted and experimental values ranged from 0.5% to 12.3%, indicating acceptable agreement and confirming the adequacy of the developed response surface models. The selected extraction conditions also fell within the experimental domain of the design, thereby supporting the reliability of the optimization procedure. Moreover, the experimental values obtained under the selected global extraction condition were comparable to those achieved under the individual optimum conditions for each response, indicating that the use of a single procedure did not substantially compromise extraction performance.

### 2.3. Method Validation for Caffeine Determination

The HPLC method showed linearity over the evaluated concentration range (10–250 µg mL^−1^), with coefficients of determination (R^2^) ≥ 0.9996. The limits of detection (LOD) and quantification (LOQ) were 5.5 and 18.2 µg mL^−1^, respectively.

Recovery values ranged from 94.5% to 95%, while intra-day and inter-day precision values ranged from 0.2% to 1.0% and 0.2% to 2.0%, respectively. The validation parameters are summarized in Table 3.

### 2.4. Chemical Composition of Coffee Samples

Table 4 presents the chemical characterization of the 21 coffee samples analyzed under the optimal extraction conditions. The table lists the values of TPC, TFC, ABTS, DPPH, and caffeine content for the analyzed samples.

TPC values ranged from 44 to 71.3 mg GAE g^−1^, whereas TFC values ranged between 30.8 and 71.2 mg QE g^−1^. The antioxidant capacity values determined via the ABTS assay ranged from 226 to 360 µmol TE g^−1^, whereas those determined via the DPPH assay ranged from 226 to 330 µmol TE g^−1^. Considerable variation was observed among the analyzed coffee samples. Consistently, *C. canephora* samples exhibited higher TPC, TFC, ABTS, and DPPH values than *C. arabica* samples.

Caffeine content ranged from 9.8 to 13.7 mg g^−1^ in *C. arabica* and from 21.2 to 29.4 mg g^−1^ in *C. canephora*. Consistently, higher caffeine levels were observed in *C. canephora* samples. Sample No. 21 exhibited the highest caffeine content among all samples.

### 2.5. Correlation Analysis

**Figure 2:**
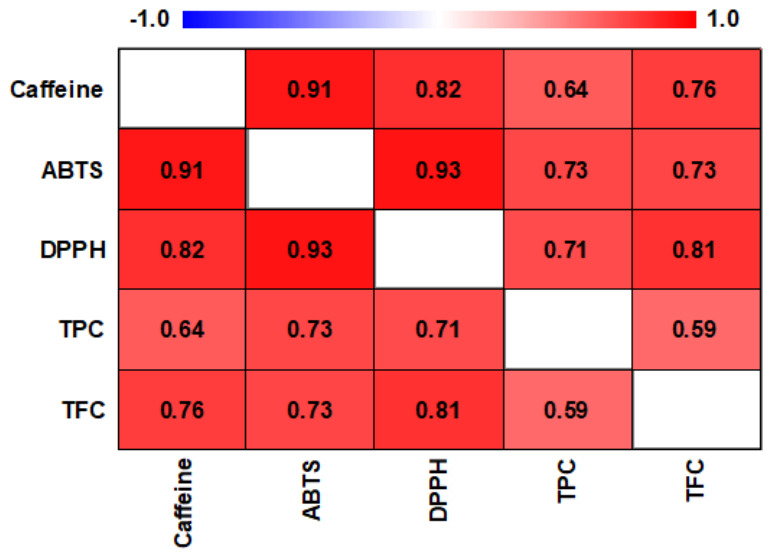
Pearson correlation heatmap showing relationships among caffeine content, total phenolic content (TPC), total flavonoid content (TFC), and antioxidant activity (ABTS and DPPH) in coffee samples (*n* = 21). All correlations were statistically significant (*p* < 0.01).

Pearson correlation analysis was performed to evaluate the relationships among phenolic composition, antioxidant activity, and caffeine content (Figure 2).

A strong positive correlation was observed between ABTS and DPPH antioxidant assays (r = 0.93). Caffeine showed positive correlations with ABTS (r = 0.91) and DPPH (r = 0.82). Moderate correlations were observed between TPC and antioxidant activity (TPC–ABTS, r = 0.73; TPC–DPPH, r = 0.71). TFC was positively correlated with DPPH (r = 0.81) and ABTS (r = 0.73), and was moderately correlated with caffeine (r = 0.64) and TPC (r = 0.59).

### 2.6. Multivariate Analysis of Coffee Samples

**Figure 3:**
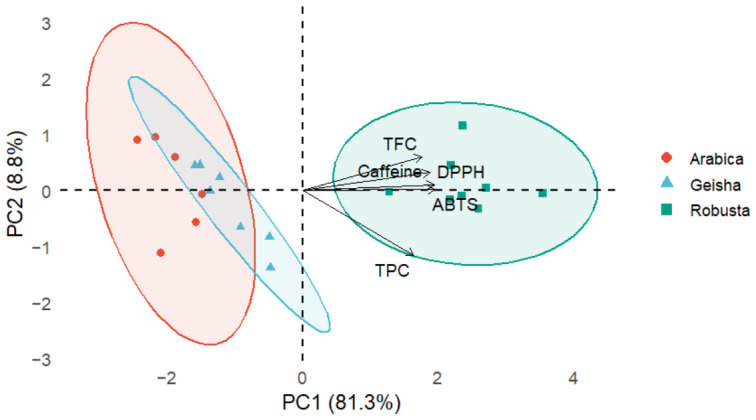
Principal component analysis (PCA) biplot of coffee samples based on total phenolic content (TPC), total flavonoid content (TFC), antioxidant capacity (ABTS and DPPH), and caffeine content. PC1 explained 81.3% of the total variance, whereas PC2 accounted for 8.8%.

Principal component analysis (PCA) was applied to evaluate the relationships among the analyzed coffee samples. The first two principal components explained 90.5% of the total variance, with PC1 accounting for 82.6% and PC2 for 7.9%. The PCA biplot (Figure 3) showed a clear separation between *C. arabica* and *C. canephora* samples along PC1. *C. canephora* samples were grouped on the positive side of PC1, whereas *C. arabica* samples were located on the negative side.

**Figure 4:**
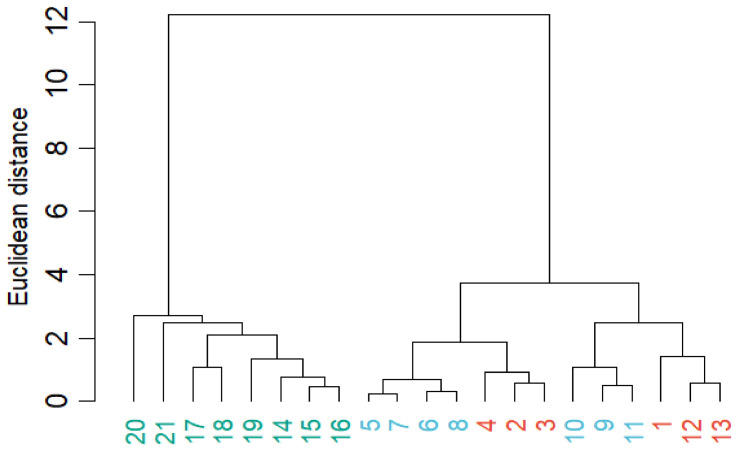
Hierarchical cluster analysis (HCA) dendrogram of coffee samples based on Euclidean distance using Ward’s linkage method. Sample labels are color-coded by coffee type: robusta (green), Geisha (light blue), and arabica (red).

Hierarchical cluster analysis (HCA) (Figure 4) showed a clear separation of *C. canephora* samples from the *C. arabica* group, which is in agreement with the PCA results.

**Figure 5:**
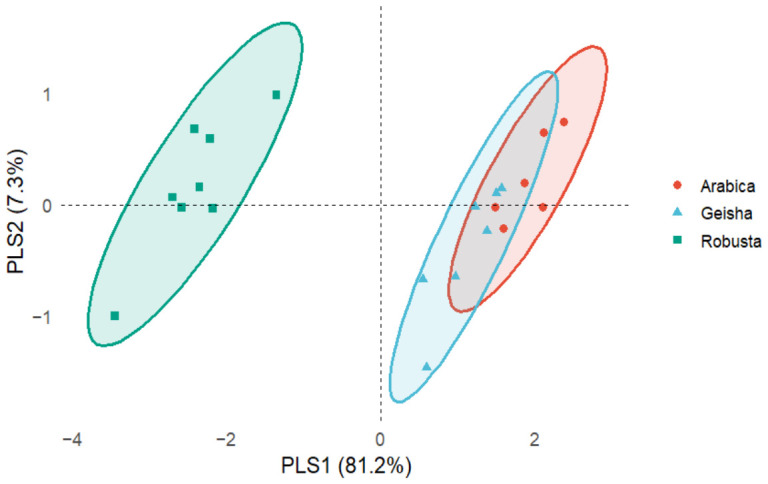
Partial least squares discriminant analysis (PLS-DA) score plot based on TPC, TFC, ABTS, DPPH, and caffeine content of roasted coffee samples. Colored ellipses represent the 95% confidence regions.

To complement the unsupervised analyses, a supervised PLS-DA model was developed using TPC, TFC, ABTS, DPPH, and caffeine data. The score plot showed complete separation between *C. arabica* and *C. canephora* samples (Figure 5). Cross-validation (5-fold, 10 repetitions) resulted in a zero classification error and a balanced error rate of zero, with one latent component selected as optimal. According to the VIP scores, ABTS (1.126), caffeine (1.097), and DPPH (1.074) showed the highest contribution values, followed by TFC (0.897) and TPC (0.754).

## 3. Discussion

The application of the RSM allowed for an evaluation of the combined effects of extraction variables on the recovery of phenolic compounds and antioxidant activity in coffee samples. The results demonstrated that temperature, ethanol concentration, and extraction time significantly influenced the extraction process, with temperature identified as the most relevant factor. The optimal temperature (~76 °C) can be explained by the increased solubility and mass transfer of phenolic compounds, enhancing their release from the coffee matrix. However, excessive temperatures may promote the degradation of thermolabile compounds [19]. Solvent composition also played a critical role, with the optimal ethanol concentration (~44%) indicating the importance of solvent polarity, allowing for the extraction of both hydrophilic and moderately hydrophobic compounds [19]. The extraction time contributed to compound recovery by facilitating diffusion [20], although prolonged times did not significantly improve yields once equilibrium was reached. Similar extraction systems based on hydroethanolic solvents and ultrasound-assisted methods have been widely reported for the efficient recovery of phenolic compounds from coffee matrices, which are known to contribute to antioxidant activity measured via ABTS and DPPH assays [21,22].

Under the optimized extraction conditions, the analyzed coffee samples showed considerable variation in TPC and TFC, reflecting differences in species and sample characteristics. *C. canephora* samples exhibited higher TPC values than *C. arabica*, consistent with previous reports indicating higher phenolic compound concentrations in Robusta coffee. For example, Hęś et al. [23] observed higher TPC values in Robusta coffee silverskin ethanol extracts (44.3–79.9 mg GAE g^−1^ dry matter) compared with Arabica silverskin extracts (34.3–47.5 mg GAE g^−1^ dry matter). Orpong et al. [8] reported TPC values of approximately 55.19 mg GAE g^−1^ and 38.6 mg GAE g^−1^ for Robusta and Arabica samples, respectively. A similar trend has also been reported for TFC, with higher flavonoid contents frequently observed in Robusta than in *C. arabica*, reinforcing the species-related differences found in the present study [24]. These differences can be attributed to variations in species, origin, and processing conditions [8,25,26]. Although the roast degree and fermentation conditions are known to influence the phenolic composition of coffee, both species covered overlapping roasting ranges, and fermentation-related variability was observed across samples in the present study. Therefore, the differences observed in TPC and TFC can be attributed mainly to intrinsic compositional differences between *C. arabica* and *C. canephora*.

Likewise, the antioxidant capacity values determined via ABTS and DPPH assays in the present study are consistent with those reported in the literature for both *C. arabica* and *C. canephora.* In agreement with previous findings, *C. canephora* exhibited higher antioxidant capacity than *C. arabica,* which can be largely attributed to the higher concentrations of phenolic compounds typically found in this species [23,27]. These findings reinforce the role of phenolic composition as a primary factor influencing the antioxidant capacity of coffee, a relationship widely reported in food systems [28,29].

The HPLC method showed satisfactory analytical performance in terms of linearity, precision, and recovery, supporting the reliability of the caffeine concentrations determined in the analyzed samples. In addition to phenolic compounds, caffeine levels differed significantly between the two coffee species analyzed. The *C. canephora* samples exhibited higher caffeine concentrations than *C. arabica*, which is consistent with previous compositional studies [8,30]. Reported caffeine concentrations in roasted Arabica coffee typically range from 4 to 11 mg g^−1^, whereas *C. canephora* commonly exhibits values between 6 and 15 mg g^−1^, depending on the origin and roasting degree [8,23]. In the present study, caffeine concentrations ranged from 9.8 to 13.7 mg g^−1^ in *C. arabica* samples and from 21.2 to 29.4 mg g^−1^ in *C. canephora* samples, showing a clear separation between the two species. These differences are mainly attributed to species-specific metabolic characteristics [31] while the influence of processing conditions appears limited, with only minor variations having been reported across different roasting conditions [32].

It is worth noting that sample No. 21 exhibited the highest caffeine content among the analyzed samples, but this was not accompanied by a proportional increase in phenolic compounds or antioxidant activity (Table 4). This distinct profile highlights variability within the *C. canephora* group. The reasons for this behavior cannot be fully established; however, it may be an interesting target for future studies focused on compositional variability in Robusta coffee.

The relationship between phenolic composition and antioxidant activity observed in this study was further supported by the correlation analysis. Significant positive correlations were found between TPC and antioxidant activity (r = 0.73 for ABTS and r = 0.71 for DPPH), indicating that phenolic compounds play an important role in determining the antioxidant capacity of coffee extracts. Notably, TFC showed a stronger correlation with DPPH activity (r = 0.81) than TPC, suggesting that flavonoids may contribute more prominently to radical scavenging activity. This behavior can be attributed to the structural characteristics of flavonoids, which enable efficient hydrogen atom or electron donation [33]. These findings are consistent with previous studies reporting that phenolic acids and flavonoids are major contributors to the antioxidant properties of coffee, and that their activity depends on their chemical structures and reactivity with respect to different radical species [28,29]. For example, Wu et al. [34] have reported significant correlations between phenolic compounds and antioxidant activity (TPC–ABTS, 0.75; TPC–DPPH, 0.79). Likewise, Cueva et al. [27] obtained a strong positive association between TPC and ABTS (0.66). In the same study, TFC showed a similar pattern, with a high correlation with ABTS (0.78) but a lower correlation with DPPH (0.33).

The correlation analysis also revealed strong positive relationships between caffeine and antioxidant activity (caffeine–ABTS r = 0.91; caffeine–DPPH r = 0.82). Although phenolic compounds are considered the primary contributors to coffee antioxidant activity, these results suggest that caffeine may also be associated with the antioxidant capacity of coffee extracts. This association may reflect both the intrinsic antioxidant properties of caffeine and its co-occurrence with phenolic compounds in coffee matrices, which can lead to synergistic effects. Previous studies have reported variable relationships between caffeine and antioxidant activity. Orpong et al. [8] found strong positive correlations between caffeine and DPPH activity in both Arabica (0.72) and Robusta (0.75) samples, whereas Cueva et al. [27] reported only a weak association between caffeine and DPPH (0.18).

It is important to note that samples from each coffee species were obtained using different processing methods, which may influence their chemical composition [9,35]. However, despite this intra-species variability, a clear separation between *C. arabica* and *C. canephora* samples was observed, indicating that species-related differences were the dominant factor driving sample discrimination.

Within the *C. arabica* group, the observed compositional variability may be associated with cultivar genetics, environmental conditions, and post-harvest processing methods. Factors such as altitude and fermentation processes can significantly influence the chemical composition, antioxidant properties, and phenolic profile of coffee beans, thereby contributing to the variability observed within this species [25,27,36,37]. In this context, specialty varieties such as Geisha may exhibit differences in their chemical profiles, reflecting both genetic characteristics and specific processing conditions.

Multivariate statistical analysis further supported the compositional differences observed between the samples. The PCA results revealed a clear separation between *C. arabica* and *C. canephora* samples along PC1, indicating that the selected chemical parameters effectively discriminated between the two species. All evaluated variables, including caffeine, TPC, TFC, ABTS, and DPPH, were strongly associated with PC1, suggesting that this axis captures a general gradient of bioactive compounds contributing to species differentiation. Although sample No. 21 showed a more extreme position within the *C. canephora* group, this did not alter the overall clustering pattern, indicating that the observed species differentiation was robust to variability among individual samples. These results highlight the usefulness of combining chemical markers with multivariate approaches for discriminating coffee species, which is consistent with recent studies based on phenolic profiling and chemometric classification [9,10].

The hierarchical cluster analysis (HCA) results were consistent with the PCA results, with *C. canephora* samples grouped into a clearly independent cluster. In contrast, *C. arabica* and Geisha samples were distributed within related sub-clusters, indicating compositional similarity among these groups. The absence of a distinct Geisha cluster suggests that, under the evaluated chemical parameters, this cultivar shares a profile closer to other *C. arabica* samples than to *C. canephora.* The presence of sub-clusters within the *C. arabica* group also suggests additional variability associated with cultivar characteristics.

The supervised PLS-DA model further confirmed the strong compositional differences between coffee species observed via PCA and HCA. Cross-validation showed complete classification accuracy, indicating robust predictive discrimination based on the evaluated chemical variables. According to the VIP scores, ABTS, caffeine, and DPPH were the variables with the highest contributions to the model, suggesting that antioxidant-related parameters, together with caffeine, play a major role in species differentiation.

Although these results demonstrate strong classification potential, the models should be considered exploratory, due to the limited sample size, and should be validated on larger and more diverse datasets. Future studies by our research group will focus on expanding the sample set and refining supervised classification models to develop rapid, practical tools for coffee authentication.

The findings of this study have important implications for coffee authenticity and fraud detection. The compositional differences observed between *C. arabica and C. canephora*, particularly in caffeine and antioxidant-related variables, support their use as indicators of potential adulteration. Their contributions to multivariate discrimination (PCA, HCA, and PLS-DA) suggest that mixtures of Arabica with Robusta could be identified through shifts in chemical profiles. However, experimental validation using samples intentionally spiked with known proportions of Robusta is required to confirm the sensitivity and practical applicability of this approach. Such studies represent an important direction for future research.

It is also important to consider that these chemical parameters may be influenced by factors such as cultivation conditions, processing methods, and roasting degree, which can introduce variability in the chemical profiles of coffee samples. This variability may limit the use of individual markers for adulteration detection, particularly in complex mixtures. Nevertheless, the multivariate approach applied in this study enables the integration of multiple variables, enhancing the robustness of the analysis and improving discrimination between coffee species despite inherent variability.

These results demonstrate that the combined evaluation of phenolic composition, antioxidant activity, and caffeine content, together with multivariate statistical analysis, provides a robust and reliable framework for distinguishing coffee species and assessing compositional variability.

## 4. Materials and Methods

### 4.1. Chemicals and Reagents

All chemicals and reagents used in this study were of analytical grade and were obtained from reputable suppliers. Sulfuric acid (H_2_SO_4_), hydrochloric acid (HCl), potassium chloride (KCl), sodium acetate (CH_3_COONa), sodium carbonate (Na_2_CO_3_), sodium nitrite (NaNO_2_), ethanol, methanol, acetic acid, ethyl acetate, hexane, and chloroform were purchased from Merck (Darmstadt, Germany) and PanReac (Darmstadt, Germany). Aluminum chloride (AlCl_3_), ferric chloride (FeCl_3_), sodium hydroxide (NaOH), and the Folin–Ciocalteu reagent, as well as 2,2-diphenyl-1-picrylhydrazyl (DPPH), 2,2′-azinobis-(3-ethylbenzothiazoline-6-sulfonic acid) (ABTS), gallic acid, quercetin, caffeine, and Trolox, were obtained from Sigma-Aldrich (St. Louis, MO, USA). Ultrapure water was used in all experiments.

### 4.2. Raw Materials

Coffee samples were collected from the main coffee-producing regions of Panama, including Boquete and Renacimiento (Chiriquí Province) and Penonomé (Coclé Province). Notably, Boquete is renowned worldwide for its production of high-quality *C. arabica*. A total of 21 samples were analyzed, including *C. arabica* (*n* = 13) and *C. canephora* (*n* = 8).

The samples included both green and roasted coffee beans from different varieties of *C. arabica*, cultivated at altitudes between 1300 and 1600 m, and *C. canephora* (Robusta), grown at altitudes between 450 and 700 m. These represent the two most commercially relevant species in Panama.

The *C. arabica* varieties analyzed included Caturra, Pacamara, Venecia, Bourbon amarillo, and Geisha, depending on availability at the time of sampling. *C. canephora* (Robusta) samples were also obtained from the above regions. All samples were obtained from different local producers and correspond to independent coffee lots from the 2025 harvest season. Subsamples from each lot were used for analytical measurements to ensure reproducibility. The samples were selected to represent the diversity of commercially available coffees in Panama.

### 4.3. Sample Processing and Preparation

Coffee samples were collected as either green or roasted beans. To ensure comparability among samples, all green coffee samples were subjected to a controlled roasting process prior to analysis; therefore, all samples were analyzed in their roasted form. Roasting was carried out using a coffee bean roasting device for 5 min, followed by a 5 min cooling cycle, reaching temperatures between 200 and 230 °C, in accordance with local processing practices [17]. According to SCA roast color values (39.8–77.5 UA for Arabica and 44.4–71.0 UA for Robusta), the samples represented a broad roasting range from light to dark roast.

After roasting, all samples were dried at 40 °C, ground, sieved to <200 μm, and stored under vacuum in the dark until further analysis, following previously reported procedures [38].

### 4.4. Optimization of Phenolic Extraction and Antioxidant Capacity

Phenolic and flavonoid compounds were extracted via ultrasound-assisted extraction using hydroethanolic solutions, and the conditions were optimized using the RSM. Briefly, 0.5 g of dried coffee was sonicated with 10 mL of hydroethanolic solvent in an ultrasonic bath (Elma S60H, Singen, Germany) under controlled temperature and time conditions. The effects of temperature (22.9–77.1 °C), ethanol concentration (22.9–77.1%), and extraction time (6.5–33.5 min) on TPC, TFC, and antioxidant capacity, evaluated via ABTS and DPPH assays, were investigated using a circumscribed central composite design, including factorial points, axial (star) points, and replicated center points. Second-order polynomial models were fitted to the experimental data using multiple linear regression, and model significance and adequacy were assessed via analysis of variance (ANOVA) at a 95% confidence level. The experimental design, model fitting, and optimization were performed using MODDE^®^ software (version 12, Umetrics, Umeaa, Sweden).

### 4.5. Phenolic Compound and Flavonoids Content Evaluation

TPC was determined using the Folin–Ciocalteu assay, as described previously [39]. Different diluted extracts (500 μL) were mixed with 2500 μL of Folin–Ciocalteu reagent (0.2 mol L^−1^) and allowed to stand for 5 min. Then, 2 mL of 75 g kg^−1^ sodium carbonate solution was added, and the mixture was incubated in the dark at 25 °C for 2 h. The absorbance of the solutions was measured at 754 nm using a visible light spectrophotometer (Genesys 10S, Thermo Scientific, Waltham, MA, USA). A calibration curve was prepared using gallic acid solution (0–12 µg mL^−1^), and the results are expressed as milligrams of GAE per gram of dry mass.

TFC was determined spectrophotometrically using the AlCl_3_ colorimetric method [40] with modifications. Briefly, 50 µL of the extract was diluted to 5 mL with distilled water, followed by the addition of 0.3 mL of 50 g L^−1^ NaNO_2_. After 5 min, 0.3 mL of 100 g L^−1^ AlCl_3_ was added to the mixture. Then, 1 mol L^−1^ sodium hydroxide (NaOH) was added, followed by distilled water to bring the total solution volume to 10 mL. After 15 min, the absorbance of the solution was measured at 374 nm using a UV spectrophotometer (Genesys 10S; Thermo Scientific, USA). A calibration curve was constructed using quercetin solutions (0–15 µg mL^−1^), and the flavonoid content is expressed as milligrams of QE per gram of dry mass.

### 4.6. Determination of Antioxidant Activity Using the ABTS and DPPH Assays

The ABTS assay was performed as described by Re et al. [41]. ABTS radical cations were generated by mixing equal portions of 7 mM ABTS solution and 2.45 mM potassium persulfate solution. The mixture was incubated in the dark at 25 °C for 16 h and diluted with 96% ethanol until the absorbance at 754 nm reached 0.7 ± 0.02. For the assay, 1900 µL of the ABTS•+ solution was mixed with 100 µL of diluted extract, and the mixture was incubated at room temperature in the dark for 10 min. The absorbance of the samples was measured at 754 nm using a visible light spectrophotometer (Genesys 10S, Thermo Scientific, USA).

The DPPH assay was conducted as described by Monrroy et al. [42] and Thaipong et al. [43]. DPPH radical solution was prepared by dissolving 13 mg of DPPH in 100 mL of methanol. To achieve an absorbance of 0.70 ± 0.02 at 515 nm, 10 mL of this solution was diluted with 45 mL of methanol. For the assay, 100 µL of the extract was mixed with 2900 µL of DPPH solution and incubated in the dark at room temperature for 30 min. The absorbance was measured at 515 nm. DPPH scavenging activity was calculated by comparison with a standard curve of Trolox (40 and 600 mmol L^−1^ Trolox). The results are expressed as µmol TE per gram of dry mass (µmol TE g^−1^).

### 4.7. Caffeine Quantification by HPLC

Caffeine was extracted from the coffee samples using a procedure adapted from Lemos et al. [21], with minor modifications. Briefly, 100 mg of ground coffee was extracted with 5 mL of ultrapure water at 95 °C for 10 min, followed by the addition of 2.5 mL of methanol and a second extraction performed in an ultrasonic bath (80 kHz) for 20 min at 40 °C. The extracts were centrifuged at 2500 rpm for 5 min, and the supernatant was transferred to a 10 mL volumetric flask. The solid residue was subsequently re-extracted with 1.0 and 1.5 mL of methanol, centrifuging after each step under the same conditions, and the supernatants were combined with the first extract. The combined extracts were brought to a final volume of 10 mL. Prior to HPLC analysis, all extracts were filtered through a 0.22 μm membrane and transferred to HPLC vials. Due to the high solubility of caffeine in aqueous and hydroalcoholic systems, efficient extraction can be achieved under these conditions.

Caffeine was analyzed using an HPLC system (Shimadzu LC-2040C, Shimadzu, Kyoto, Japan) equipped with a UV detector set at 274 nm and a Poroshell 120 EC-C18 column (100 mm × 4.6 mm, 2.7 μm, Agilent Technologies, Santa Clara, CA, USA) maintained at 25 °C. The mobile phase consisted of 1% acetic acid in water (pH 2.8, solvent A) and methanol (solvent B), employing a gradient elution of 90% A/10% B (0–5 min), 70% A/30% B (5–15 min), and 50% A/50% B (15–25 min), at a flow rate of 0.8 mL min^−1^ and an injection volume of 10 μL. Quantification was performed using an external calibration curve prepared from caffeine standard solutions in 80% (*w*/*w*) methanol over the concentration range of 10–250 μg mL^−1^.

The method was validated in terms of linearity, LOD and LOQ, precision, and accuracy. LOD and LOQ were calculated based on the standard deviation of the response and the slope of the calibration curve (3.3 σ/S and 10 σ/S, respectively). Precision was evaluated in terms of repeatability (intra-day) and intermediate precision (inter-day), and accuracy was assessed using spiked coffee samples at three spiking levels (corresponding to 50%, 100%, and 150% of the initial caffeine content) with four replicates.

### 4.8. Statistical and Multivariate Data Analysis

Multivariate statistical analyses were performed to explore patterns in the chemical data and evaluate the potential of the measured variables as markers for sample differentiation. A data matrix was constructed from the concentrations of total phenolic content (TPC), total flavonoid content (TFC), antioxidant capacity determined via ABTS and DPPH assays, and caffeine content for all coffee samples. Prior to analysis, all variables were autoscaled (unit-variance scaling) to ensure equal contribution from each parameter.

PCA was applied as an unsupervised method to explore natural groupings among samples based on coffee species (*C. arabica* and *C. canephora*). Hierarchical cluster analysis (HCA) was used to evaluate sample similarity based on Euclidean distances and Ward’s linkage.

In addition, partial least squares discriminant analysis (PLS-DA) was performed as a supervised classification approach using the same autoscaled variables. For this analysis, samples were grouped into two classes: *C. arabica* (including Geisha samples) and *C. canephora.* Model performance was evaluated via repeated 5-fold cross-validation (10 repetitions), and variable importance was assessed using Variable Importance in Projection (VIP) scores.

PCA, HCA, and PLS-DA analyses were performed using the R software (version 4.5.3; R Core Team, Vienna, Austria), with the PLS-DA model implemented using the mixOmics package.

Pearson correlation analysis was performed to evaluate the relationships among phenolic composition (TPC and TFC), antioxidant activity (ABTS and DPPH), and caffeine content. Correlation coefficients (r) were calculated using the Statgraphics Centurion software (version 18; Statgraphics Technologies, Inc., The Plains, VA, USA), and the results were visualized as a correlation heatmap.

Paperpal was used for grammar checking, editing, and paraphrasing.

## 5. Conclusions

Optimization of ultrasound-assisted hydroethanolic extraction using the RSM enabled the efficient recovery of phenolic compounds and antioxidant constituents from coffee samples. Selected extraction conditions, established from the response surface optimization, yielded suitable levels of total phenolic content, total flavonoid content, and antioxidant activity, as determined through ABTS and DPPH assays. Chemical characterization revealed considerable variability in phenolic composition, antioxidant capacity, and caffeine content among the analyzed samples. In general, *C. canephora* exhibited higher levels of phenolic compounds, antioxidant activity, and caffeine than *C. arabica*. Multivariate statistical analysis, including PCA, HCA, and supervised PLS-DA, revealed a clear separation of *C. canephora* samples, whereas *C. arabica* (including Geisha cultivars) exhibited partial overlap, reflecting compositional variation among Arabica cultivars. The results demonstrate that phenolic composition, antioxidant activity, and caffeine content can serve as reliable chemical markers for distinguishing *C. arabica* from *C. canephora*. In the Panamanian context, these findings are particularly relevant for supporting the authentication, quality control, and added-value differentiation of nationally produced specialty coffees. The integration of optimized extraction procedures, chemical characterization, and multivariate analysis provides a robust analytical approach for coffee classification, authenticity assessment, and quality control of coffee products.

## Figures and Tables

**Table 1:** Central composite design matrix and observed responses for ultrasound-assisted hydroethanolic extraction.

Run	Temperature (°C)	Ethanol (%)	Time (min)	TPC (mg GAE g^−1^)	TFC (mg QE g^−1^)	DPPH (µmol TE g^−1^)	ABTS (µmol TE g^−1^)
1	30	30	10	30.9	18.9	149.8	161.4
2	70	30	10	43.6	26.0	193.1	221.8
3	30	70	10	27.9	15.2	121.9	141.5
4	70	70	10	41.1	21.9	166.7	204.5
5	30	30	30	41.1	21.3	176.4	195.3
6	70	30	30	53.8	24.9	210.8	250.0
7	30	70	30	35.4	19.8	141.8	180.1
8	70	70	30	49.9	24.7	191.5	248.8
9	22.9	50	20	40.0	19.4	168.2	182.4
10	77.1	50	20	55.5	27.1	222.7	245.7
11	50	22.9	20	46.4	22.7	182.2	227.6
12	50	77.1	20	38.5	19.1	142.7	201.0
13	50	50	6.5	33.8	18.0	151.5	188.5
14	50	50	33.5	48.0	23.9	219.2	253.8
15	50	50	20	48.3	22.8	202.7	247.8
16	50	50	20	46.6	24.0	195.1	230.8
17	50	50	20	49.4	23.3	208.1	244.4

**Table 2:** Parameters of the fitted response surface models.

Response	R^2^	Adj R^2^	Model *p*-Value	Lack of Fit *p*-Value	RSD (%)
TPC	0.99	0.98	<0.001	0.787	1.1
CFT	0.96	0.93	<0.001	0.358	0.8
DPPH	0.95	0.93	<0.001	0.444	8.0
ABTS	0.97	0.96	<0.001	0.775	7.2

**Table 3:** Validation parameters for caffeine determination using HPLC.

Spiking Level (%)	Found (mg g^−1^)	Recovery (%)	Intra-Day RSD (%)	Inter-Day RSD (%)
50	16.8 ± 0.1	94.5 ± 0.8	0.8	0.9
100	21.7 ± 0.1	94.8 ± 0.2	0.2	0.8
150	26.9 ± 0.2	95 ± 1	1	2

Note: Spiking levels (50%, 100%, and 150%) were calculated based on the initial caffeine content of the sample (11.66 ± 0.03 mg g^−1^).

**Table 4:** Chemical composition of *C. arabica* and *C. canephora* samples analyzed from local producers in Panama and analyzed under optimal extraction conditions.

No.	Species	Variety	Process	TPC (mg GAE g^−1^)	TFC (mg QE g^−1^)	ABTS (µmol TE g^−1^)	DPPH (µmol TE g^−1^)	Caffeine (mg g^−1^)
*C. arabica*								
1	*C. arabica*	Caturra/Catuai	Washed	57.4 ± 0.5	30.8 ± 0.5	241 ± 1	227 ± 2	12.3 ± 0.4
2	*C. arabica*	Venecia	Natural	44 ± 1	44 ± 2	236 ± 4	246 ± 3	9.80 ± 0.04
3	*C. arabica*	Bourbon amarillo	Fermented	48 ± 1	46 ± 2	239 ± 2	243 ± 2	9.8 ± 0.1
4	*C. arabica*	Pacamara L35	Fermented	44 ± 1	42 ± 1	226 ± 1	226 ± 1	12 ± 1
5	*C. arabica*	Geisha Perla blanca	Fermented	50.1 ± 0.6	45.7 ± 0.8	237.4 ± 0.4	249 ± 0	11.6 ± 0.2
6	*C. arabica*	Geisha	Washed	52.6 ± 0.2	46 ± 2	235 ± 1	263 ± 2	12.30 ± 0.06
7	*C. arabica*	Geisha	Washed	50.3 ± 0.4	46 ± 2	232 ± 1	256 ± 2	11.6 ± 0.1
8	*C. arabica*	Geisha	Washed	54 ± 2	45 ± 1	228 ± 3	260 ± 2	11.48 ± 0.02
9	*C. arabica*	Geisha	Washed	62 ± 1	45 ± 1	265 ± 1	265 ± 1	12.4 ± 0.7
10	*C. arabica*	Geisha	Washed	67.4 ± 0.5	46.9 ± 0.5	240.8 ± 0.3	254 ± 2	12.4 ± 0.4
11	*C. arabica*	Geisha	Honey	60 ± 1	45 ± 2	250 ± 3	252 ± 0	12.1 ± 0.7
12	*C. arabica*	Caturra	Natural	54 ± 1	43.1 ± 0.8	239 ± 4	238 ± 4	13.5 ± 0.3
13	*C. arabica*	Caturra	Washed	57 ± 0	39.7 ± 0.7	236 ± 2	232 ± 1	13.7 ± 0.42
*C. canephora*								
14	*C. canephora*	Robusta	Natural	66 ± 2	60 ± 1	360 ± 4	306 ± 3	24.3 ± 0.5
15	*C. canephora*	Robusta	Not specified	65 ± 1	56 ± 3	356 ± 5	310.8 ± 0.1	22.4 ± 0.1
16	*C. canephora*	Robusta	ASD	67 ± 5	56 ± 2	360.1 ± 0.2	324 ± 3	21.6 ± 0.3
17	*C. canephora*	Robusta	Honey	59 ± 1	45 ± 3	348 ± 1	302 ± 1	21.63 ± 0.06
18	*C. canephora*	Robusta	Honey	63 ± 1	50 ± 2	357 ± 11	330 ± 1	21.8 ± 0.3
19	*C. canephora*	Robusta	Not specified	62 ± 2	61 ± 2	317 ± 14	326 ± 4	21.2 ± 0.3
20	*C. canephora*	Robusta	Not specified	71.3 ± 0.7	71.2 ± 0.4	353 ± 2	329 ± 2	21.8 ± 0.3
21	*C. canephora*	Robusta	Not specified	59 ± 5	63.5 ± 0.4	323 ± 5	289 ± 5	29.40 ± 0.01

Note: Process: Washed = washed process; Natural = dry process; Honey = semi-dry process; Fermented = extended or controlled fermentation; ASD = anaerobic slow drying. Not specified indicates that detailed processing information was not available. All values were determined in triplicate and are expressed as mean ± standard deviation.

## Data Availability

Data are contained within the article.

## Abbreviations

The following abbreviations are used in this manuscript:

ABTS	2,2′-azinobis-(3-ethylbenzothiazoline-6-sulfonic acid)
ANOVA	Analysis of variance
DPPH	2,2-diphenyl-1-picrylhydrazyl
GAE	Gallic acid equivalents
HCA	Hierarchical cluster analysis
HPLC	High-performance liquid chromatography
PCA	Principal component analysis
QE	Quercetin equivalents
RSD	Relative standard deviation
RSM	Response surface methodology
TE	Trolox equivalents
TFC	Total flavonoid content
TPC	Total phenolic content
